# Potential of Surface Enhanced Raman Spectroscopy (SERS) in Therapeutic Drug Monitoring (TDM). A Critical Review

**DOI:** 10.3390/bios6030047

**Published:** 2016-09-19

**Authors:** Aleksandra Jaworska, Stefano Fornasaro, Valter Sergo, Alois Bonifacio

**Affiliations:** Department of Engineering and Architecture, University of Trieste, Via Valerio 6a, 34127 Trieste, Italy; ajaworska@units.it (A.J.); sfornasaro@units.it (S.F.); sergo@units.it (V.S.)

**Keywords:** SERS, TDM, drug monitoring, Individualized cancer chemotherapy

## Abstract

Surface-Enhanced Raman Spectroscopy (SERS) is a label-free technique that enables quick monitoring of substances at low concentrations in biological matrices. These advantages make it an attractive tool for the development of point-of-care tests suitable for Therapeutic Drug Monitoring (TDM) of drugs with a narrow therapeutic window, such as chemotherapeutic drugs, immunosuppressants, and various anticonvulsants. In this article, the current applications of SERS in the field of TDM for cancer therapy are discussed in detail and illustrated according to the different strategies and substrates. In particular, future perspectives are provided and special concerns regarding the standardization of self-assembly methods and nanofabrication procedures, quality assurance, and technology readiness are critically evaluated.

## 1. Introduction

### 1.1. Therapeutic Drug Monitoring (TDM)

Therapeutic drug monitoring (TDM) has been used in clinical practice since the beginning of the 1970s, and it is now routinely required for a small fraction of drugs (~30) used in pharmacotherapy [1]. For these drugs (e.g., antiepileptics, immunosuppressants, anti-HIV agents, anticonvulsants, some antibiotics, and some cytotoxic drugs), such monitoring is essential to provide patients with effective treatment, while minimizing drug toxicity and minimizing the risk of adverse drug reactions. More recently, cancer therapy has become a burgeoning field for the development of novel analytical procedures for less frequently monitored drugs [2]. Personalized medicine has been increasingly advocated to improve the standard of care for oral/new molecularly targeted therapeutics, where side effects can be substantial and life threatening. However, TDM is seldom performed in clinical oncology due to cost/time considerations, analytical issues, and the lack of point-of-care instrumentation available [3,4,5,6].

TDM, in practice, involves assessing drug concentration in a biological matrix (most commonly plasma or serum) at a known time related to administration, and interpreting these concentrations in terms of relevant clinical parameters (target range, pharmacokinetics of the drug) [7]. Only a small fraction of prescription drugs requires TDM because, for the majority of drugs, there is a wider difference between the minimum effective concentration and the toxic concentration (therapeutic index). Thus the dose is easily adjusted by empirically assessed pharmacodynamic parameters (*titration to clinical effect*) [8,9]. TDM is required for drugs with a narrow therapeutic index when: (i) it is hard to correlate therapeutic or low toxicity levels with clinical effects alone; (ii) the relationship between the drug dose and serum concentration is highly variable and/or not predictable; (iii) there is a clear correlation between serum concentration of the drug and its therapeutic response or toxicity; (iv) serious consequences for under- or overdosing may be avoided by TDM [8,10].

Most of the analytical platforms commonly used for TDM in clinical practice rely on many different immunoassays, or on separation techniques coupled with mass spectrometry (MS). The fundamentals and application of these platforms will not be discussed in this review but have been extensively reported elsewhere [11,12]. A brief summary is reported in Table 1. In general, immunoassays are *routine methods* due to the ease of operation and speed, but they carry many limitations, such as interferences from components of matrices, drug metabolites, structurally similar drugs, as well as endogenous compounds. In addition, they are not available for all drugs currently monitored. On the other hand, *reference methods* such as liquid chromatography combined with tandem mass spectrometry (LC-MS/MS) are in some cases still the gold standard for TDM in clinical laboratories, because they are analytically more robust and relatively free from interferences. However, the analysis is time-consuming and labor-intensive, due to the extensive sample preparation and significant volume of samples needed for processing the biofluids.

Since the infrastructure required for TDM on chromatographic, mass spectrometric, and immunoassay techniques is expensive and not necessarily available in small hospitals, methods such as surface plasmon resonance (SPR) [13], localized SPR [14], electrochemical sensors [15], and quartz crystal microbalance (QCM) [16], have recently been proposed as potential TDM platforms able to provide a rapid response with limited infrastructure and sample preparation. However, the listed methods feature biofouling problems while performing the measurements, preventing their use for routine, large-scale testing. In fact, nonspecific binding of unwanted proteins and other molecules onto the sensing interface of devices can easily foul the active surface of the biosensors, thus generating overwhelming background signal and preventing the detection of target drugs [17,18]. In spite of the amount of research recently dedicated to the protection against fouling agents, this issue is still not fully resolved to date [19]. Therefore, new techniques, that are cheaper and faster than the current reference methods, are still sought which could be regularly and easily used in hospitals.

Surface-enhanced Raman spectroscopy (SERS) could also be a good candidate for TDM since, in principle, quantitative analyses of drugs in body fluids could be made within a few minutes and with comparable or smaller errors than routine TDM methods [20,21,22]. Although SERS shares with other methods (e.g., SPR, QCM) the problem of surface fouling, the higher amount of information present in SERS spectra makes it easier to exploit the full potential of multivariate data analysis (see Section 1.4) to limit the interference of non-specific binding. Additionally, portable Raman spectrometers are available nowadays, which are robust, small, and easy to use: therefore, they could be routinely used in clinical settings by non-specialized operators.

This review summarizes the prospective applications of SERS in the field of TDM for cancer therapy. We will compare the critical issues concerning preparation, sensitivity, and selectivity of proposed approaches, and we will discuss the direction presently taken by the techniques.

### 1.2. Surface Enhanced Raman Spectroscopy (SERS)

In 1974, Fleischmann et al. [23] discovered that molecules of pyridine adsorbed on specially prepared silver surfaces produced a Raman spectrum that was several orders of magnitude more intense than expected [24]. This effect came to be known as Surface Enhanced Raman Spectroscopy (SERS). In 1977, the discovered phenomenon enabled studies on kinetic processes of amines that occured during their adsorption at the silver electrodes [25]. Increases in the intensity of the Raman signal on the order of 10^4^–10^6^ have been regularly observed, first calculated by M. Albrecht and J. Creighton with the example of pyridine at silver electrodes [26] , and the enhancement can be as high as 10^10^–10^12^ for some systems [27]. The largest contribution to the intensity amplification results from the so-called “electromagnetic mechanism”, postulated by Van Duyne as ‘an electric field enhancement’ [25], in which the electric field is locally enhanced in the vicinity of nanostructured metal surfaces that are illuminated with light resonant with the localized surface plasmon frequency of the metal structure [24]. The electromagnetic mechanism allows for the occurrence of a SERS effect even in those cases in which analytes are not directly adsorbed on the metal, but are within a few nanometers from it. Besides the electromagnetic mechanism, for molecules chemisorbed on a SERS-active surface there is also a “chemical mechanism”, which involves the creation of new molecular states because of the direct interaction with the metal, in addition to the electromagnetic effect [25,26]. The occurrence of a SERS effect has been demonstrated with many molecules and with some metals, i.e., mainly copper, silver, and gold, although minor enhancements have also been demonstrated for lithium, sodium, potassium, indium, platinum, and rhodium [24]. SERS signals strongly depend on the type of the metal surface, which influences the presence of ‘hot spots’, defined as “highly localized regions of intense local field enhancement caused by surface plasmon resonance, usually occurring within interstitial crevices in metal structures” [28].

Many techniques can be used to produce SERS substrates, i.e., metallic nanostructures capable of inducing a SERS effect [22]. Here we decided to use a general classification of the substrates: metallic nanoparticles dispersed in a liquid medium (colloidal substrates), solid non-colloidal substrates, and “hybrid” substrates (in which the two previous substrate types are used in synergy). Colloidal dispersions are the easiest to prepare but usually require partial aggregation, which can be induced by the addition of an electrolyte such as KNO_3_ or NaCl [29,30], to form SERS-effective metal nanostructures. However, nanostars or other nanoparticles with non-spheroidal shapes may show a significant SERS effect, even in the absence of aggregation.

### 1.3. Critical Issues on the Use of SERS for TDM

TDM depends heavily on the ability to measure drug concentrations in a body fluid, most commonly serum, plasma, or whole blood. In general, several intrinsic difficulties can complicate the quantification as well as identification of analytes with SERS. First of all, an absolute SERS signal is very difficult to achieve since Raman scattering intensity can fluctuate due to various experimental conditions, such as pH conditions, laser wavelength and power, and the optical alignment of the instrument. Thus, a fine control of those factors is crucial for reliable quantitative measurements [31,32]. Secondarily, qualitative variations within the SERS substrate can affect the hot spots distribution and efficiency, making it difficult to reproduce the signal even for the same sample [33]. The use of internal standards has been recently proposed as a possible solution for this problem [34,35].

While SERS is expected to provide quantification of a specific target analyte in complex biofluids, in practice several drugs can be detected and quantified in simple solutions, but the presence of other biomolecules severely interferes with the SERS spectra, making quantification in real samples a challenging task. Clinical samples are complex mixtures containing lipids and proteins that can bind to the drug of interest. The reference interval for total serum protein is 60–80 g/L [36], while target drugs are present at considerably lower concentrations (usually ng/L). Consequently, only a fraction of the drug molecules will be free to interact with the metal surface, and thus to benefit from the signal enhancement. Then, even free, unbound analytes must compete for adsorption with the sample constituents, in order to be detected. Human serum, for instance, contains more than 4000 metabolites [37], some of which have a high affinity for metal surfaces, as proven by the intense SERS signal given by serum when using metal colloids [38].

Therefore, several authors have focused on obtaining SERS signals from biological samples under controlled experimental conditions, aimed at simplifying the chemical complexity of the sample itself, in the bulk or at the metal surface [39]. This simplification could be achieved, for instance, either through the integration of SERS with separation techniques or via the functionalization of the SERS substrates with selective recognition elements, hence ensuring specificity for an analyte of interest while minimizing the interference from other biochemical species present in the sample.

Many separation technologies, like thin layer chromatography (TLC) [40] and capillary electrophoresis [41], have been coupled with SERS to separate the analytes from interfering species before SERS detection. Further improvements aimed at physically integrating many complex devices or functions in small instruments (e.g., lab-on-a-chip approaches), working with a high degree of automation, will be of tremendous importance for future clinical implementations of SERS.

An alternative way to efficiently separate the target analytes from the matrix components is the modification of the metal surface, directly or via a short linker moiety, with specific recognition elements (also called “artificial receptors”), such as macrocyclic host molecules, small peptides, or molecularly imprinted polymers (MIPs), to promote the selective surface binding of particular analytes [42].

Another issue, often underestimated, to be carefully considered in SERS measurements, is the choice of the laser wavelength. This parameter will affect results in at least three ways.

First, the laser should be able to efficiently excite the localized surface plasmons of the SERS substrates used.

Secondly, when the laser wavelength falls within an electronic transition band of the analyte, a Resonance Raman (RR) effect will add to the SERS effect, yielding a so-called surface-enhanced resonance Raman scattering (SERRS) spectrum. SERRS ensures a greater enhancement which is selective with respect to the resonant analyte, thus partially solving the problems due to spectral interference of other components. The SERRS effect can be exploited for all those drugs which absorb in the visible region, such as doxorubicin [43,44] or mitoxantrone [45]. However, biomolecules naturally present in many biofluids, such as carotenoids, also absorb in the visible region, yielding intense SERRS bands which may interfere with drug detection.

A third aspect, related to the excitation wavelength, concerns the spectral interference of fluorescence due to endogenous fluorophore species present in biofluids. All these aspects have been discussed in a recent review about SERS of biofluids [38], and since they can positively or negatively impact the results, they must be carefully considered when designing an experimental approach for a SERS quantification of a drug in biofluids.

### 1.4. Quantification with SERS: The Role of Data Analysis

To monitor the concentration of a certain drug in biofluids, it is necessary to build a model, as a calibration curve, through which it is possible to extrapolate the information about the concentration of the drug from SERS spectra. However, a typical SERS spectrum can be rather complex, consisting of a large number of variables (*p*, i.e., Raman shifts), typically on the order of 1000 or more, even when the number of samples (*n*) is relatively small (causing the so-called large *p,* mall *n* problem) [46].

In a univariate calibration, known concentrations of reference standards are assembled. Single intensities (or integrated areas) are plotted against drug concentrations and the pertinent calibration is the result of a regression, relating a measured intensity (or integrated area) value to the drug concentration [47]. Although univariate calibration is the most intuitive and simple situation, its application requires both a high signal-to-noise ratio (S/N) and an instrumental response, which depends only on the concentration of the analyte of interest. Interferences from biofluid constituents may contribute to the measured intensity at a specific Raman shift, making it arduous to differentiate an analyte-specific signal from an interfering one, when considering only one point in the data spectrum.

An alternative that may overcome most of the limitations of univariate calibration is a multivariate calibration, which uses multiple responses simultaneously (e.g., the response at a range of Raman shifts, or over the entire range collected), to calculate concentrations. Spectral data are treated using chemometric tools, mainly by the application of regression techniques. The advantage over univariate methods lies in a higher accuracy due to the inclusion of more spectral information and less noise [48,49,50].

Various methods have been developed for building a multivariate calibration model, each of which aims to simplify complex spectroscopic information. While a comprehensive description of these methods is beyond the scope of this paper, many examples of applications to a wide range of studies can be found in the literature [47,51,52]. The technique most widely used in combination with SERS is partial least squares regression (PLSR), which manages the data with more variables than samples by replacing the original variables with a few latent variables (LVs) [53]. Many popular variants of the PLSR algorithm are available and, in some works, hybrid methods which use a combination of two or more statistical tools (such as, for example, elastic net (EN) or principal component analysis (PCA)) have been employed to extract relevant chemical information before applying the PLSR [54,55].

Spectral data pre-processing procedures (involving fluorescence background/cosmic ray artefacts removal, outlier rejection, dimensionality reduction) have a strong influence on the outcome of the subsequent quantitative analysis [56]. “Appropriate” pre-processing combinations can enhance the model, increasing its accuracy and lowering the error of prediction, while “not appropriate” combinations can introduce a decline of the model performance [57]. These factors must be taken into account during the development of any multivariate data analysis.

A reliable application of SERS for TDM routine analysis requires (i) an appropriate validation of the regression model to avoid overfitting; and (ii) the establishment of *figures of merit* (FOMs) to certify the prediction ability and define the quality of the analytical method [58,59]. Concerning the first point, there is a general consensus about the fact that the only sure way to prove a model’s robustness is to use an independent test set, which contains only data from samples that were not used in the previous generation/optimization of the model [60]. However, when the number of samples is too small to construct a proper independent test set, more sophisticated methodologies using resampling/bootstrapping nested procedures, such as Repeated Double Cross-Validation [61,62], are considered as suitable alternatives. Concerning the FOMs needed for an analytical method to meet the regulatory requirements moving from the laboratory scale to the clinical level, some relevant guidelines are already available (see, for instance, 2002/657/EC [63], ISO 11843 [64], and IUPAC [65]). FOMs concepts include selectivity, specificity, linearity response, accuracy (trueness and precision), reproducibility, decision limit (CCα, former limit of detection, LOD), and detection capability (CCβ, nowadays preferred to the more known limit of quantification, LOQ). It must be noted that while the reporting of FOMs is straightforward and well defined for univariate calibrations, the evaluation of FOMs in multivariate calibrations is much more difficult and controversial [47,66].

## 2. Applications of SERS Relevant to TDM

The papers published so far reported results from measurements performed in body fluids (serum, saliva, blood, plasma, urine, vein/muscle), in surrogate matrices mimicking body fluid conditions (buffered solutions of bovine serum albumin (BSA), or human serum albumin (HSA)), and in water/inorganic solvents (Table 2). Only one publication describes experiments performed on clinical samples from patients treated with mitoxantrone [45]. For a better comparison, the papers considered in this review were grouped according to the type of the SERS substrates used (colloidal, non-colloidal, and hybrid substrates, as already mentioned).

### 2.1. Colloidal Substrates

Colloidal nanoparticles are the most commonly used substrates in SERS because of their relatively low cost and ease of preparation. To the best of our knowledge, so far there are two publications that successfully perform SERS measurements by directly adding colloidal silver nanoparticles (AgNPs) to the sample; detection of doxorubicin and paclitaxel in bovine plasma (BP [70]) and human serum albumin (HSA) solution [74], respectively.

In the case of doxorubicin [70], AgNPs were activated with sodium chloride (NaCl) and doxorubicin solutions were prepared in 1% bovine plasma. The calibration curve showing the dependence of the intensity of the SERS signal on the analyte concentration was reported in a concentration range between 100 and 750 nM. In this case, the SERS technique presents a detection limit at much higher concentrations with respect to the reference methods (e.g., for HPLC-MS and CE-LIF, LOD is around 1–2 nM). However, it is important to note that the measurements did not require any sample pre-treatment, unlike chromatographic or electrophoretic measurements. Paclitaxel detection with SERS on colloids was shown in a preliminary report, where AgNPs activated with sodium nitrate (NaNO_3_) were used as substrates in HSA solutions [74]. The lowest concentration detected of paclitaxel, at different pH values, was 10^−5^ M.

Colloidal metal nanoparticle dispersions can also be modified with additional nanostructures (achieving so-called “hybrid” nanoparticles) to enhance their SERS performance. Recently, graphene-modified surfaces have gained increasing popularity as SERS substrates due to their stability, and to the enhancement and repeatability of the SERS signal. Successful determination of 6-mercaptopurine (6-MP) in tablets was performed with the use of graphene-modified metal nanoparticles [78]. In this work, hybrids of graphene oxide (GO) and metal nanoparticles (MNPs) through the mediation of polyethyleneimine (PEI) molecules were used. For the quantification of this drug in pharmaceutical tablets, SERS results using these hybrid nanoparticles did not show significant differences when compared to the standard UV-Vis measurements, indicating the potential of the method.

Another possible approach is to modify the nanoparticles with molecules that can mediate the interaction with the analyte. Yang et al. [75] have shown that silver nanoparticles coated with β-cyclodextrin (β-CD) are more efficient as SERS substrates when compared to the citrate-reduced colloids. The authors of this study showed that the addition of β-CD improves sensitivity, time of analysis, and the linear range of the SERS response, besides making the colloids more stable toward aging. With these modified nanoparticles, 6-MP could be quantified in aqueous solutions with a LOD of 2.4 nM and a limit of quantification of 0.8 nM.

Because of the chemical complexity of biofluids, as mentioned in Section 1.3, it might be useful to combine SERS with separation techniques, such as thin layer chromatography (TLC). In a work by our own research group [40], irinotecan at the concentration of 2.27 and 27.3 μM was detected in water and in HSA solutions, respectively, after a combined deproteinization/separation step using TLC. The irinotecan solution underwent a deproteinization and separation step on the TLC plate, using a mixture of chloroform and methanol with the two-fold role of deproteinization solvent and eluent. A drop of colloid was then deposited on the TLC plate where the drug was expected according to a reference position (determined using pure irinotecan solutions). This approach, despite the advantage of combining deproteinization and separation in a single step, still had poor repeatability, related to the use of dried spots of metal colloids as substrates.

**Figure 1:**
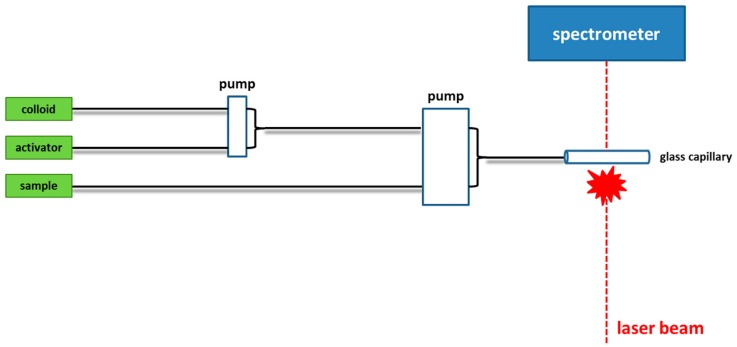
Schematic diagram of the flow cell used for quantitative analysis of mitoxantrone in clinical serum samples, adapted from [45].

Other authors reported the use of a flow system to mix samples and colloidal substrates to optimize nanoparticles aggregation for SERS detection, with the aim of increasing repeatability [31,82,83]. In these approaches the metal colloids, the aggregating agents, and the sample are pumped in a flow system (constituted by a macroscopic flow cell [45] or a microfluidic system [77]), mixed at some point, and are then measured using a Raman microscope. A schematic diagram of the flow system used in this study is shown in Figure 1.

In a pioneering work by McLaughlin et al. [45], which is still considered to be one of the most successful SERS applications of drug quantification in biofluids, quantification of mitoxantrone in real serum samples from patients was performed, showing an excellent correlation with a reference method such as HPLC. Citrate-reduced silver colloids, activated (i.e., pre-aggregated) with NaCl or poly-l-lysine, were used as the SERS substrate.

The measurements were performed in plasma and serum in the concentration range between 2.5 × 10^−9^ M and 1 × 10^−6^ M. To verify the utility of the SERS methodology for the clinical samples, serum was collected from patients treated with MTX one day before. The samples were measured by both standard HPLC and SERS, and Table 3 shows the potential of the latter as an alternative technique for TDM measurements.

Hidi et al. [80] used a similar approach, but based on a microfluidic system with a Lab-on-a-Chip set-up, to quantify methotrexate (MTX) in KOH solutions at pH ~12 with the use of silver colloid activated with KCl as the SERS substrate. As in the paper by McLaughlin et al. [45], in this work colloidal aggregation was controlled using a flow system. However, the feasibility of a microfluidic SERS chip for MTX quantitation was only demonstrated with model solutions, while its efficiency with more complex biofluids has yet to be tested. In general, controlled aggregation of the metal colloids using a flow system produced repeatable results, but at the cost of a more complex (and perhaps more expensive) setup.

Interesting quantitative applications of SERS of drugs were also reported for samples other than biofluids. For instance, Han et al. [84] reported the monitoring of metabolism of 6-MP in living cells. It turned out that after being uptaken into cells, gold nanoparticles conjugated to 6-MP were metabolized to 6-MP-ribose after 4 h and the signal drastically decreased after 16 h. A similar label-free approach for the SERS quantification of 6-MP in HeLa cells was also reported by Yang et al. [85]. Although not directly aimed at quantification, these papers show how quantitative SERS can also be applied to study drug metabolism in living biological systems.

### 2.2. Non-Colloidal Substrates

Without the problems due to colloidal stability, non-colloidal substrates offer much more possibilities for the chemical functionalization of metal surfaces with recognition elements. Such surface modifications allow for an increase in repeatability, an improvement in the analyte signal due to an increased drug concentration near the metal, as well as a more straightforward procedure, avoiding the handling of the colloidal substrates and their mixing with the analyte.

In recent years, the number of papers involving the preparation of new non-colloidal SERS substrates is rapidly increasing [86]. The large number and variety of substrates reported in the literature shows the growing interest in this topic, as well as the lack of consensus about what substrate performs better. This is caused, among other factors, by the fact that different molecules interact with substrates in a variety of ways, so that perhaps a “universal” SERS substrate, which can be generally applied to all analytes, is just an abstract concept. Indeed, different kinds of non-colloidal substrates have been used for the detection of TDM drugs, with different outcomes.

Klarite^TM^ substrates were commercially available gold-coated nanostructured silica surfaces distributed by Renishaw Diagnostics Ltd. In a recent work by Litti et al. [79], methanol solutions of sunitinib, paclitaxel, irinotecan, SN-38 (a metabolite of irinotecan), and doxorubicin (as reference) were deposited on the Klarite^TM^ substrates and dried in air at room temperature. Apart from paclitaxel, the other drugs were quantified at therapeutic concentrations using a univariate approach. This work clearly showed the feasibility of SERS quantification of these drugs, although in simple model solutions, without the complications due to the complexity of real biofluids. In this article, authors also remarked that while the density of hot spots is crucial for the determination of the limit of quantification, the homogeneity with which such hot spots are distributed onto the surface is related to repeatability: both these aspects are important while designing new SERS substrates.

Another promising type of solid SERS substrate employed for drug quantification with SERS was recently obtained using UV-assisted nano-imprint lithography (UV-NIL) [81]. With this substrate, the authors reported SERS spectra of water solutions of paclitaxel down to nanomolar concentrations. UV-NIL substrates were covered with a monolayer of BSA, which in this case was used as a recognition element for binding the drug close to the metal. The SERS intensity of a band in the C-H stretching region showed a linear dependence on the concentration of paclitaxel between 1.2 × 10^−9^ M and 11.9 × 10^−6^ M. The authors of this study remarked that the LOD obtained with this approach was similar to that achieved with LC-MS-MS, and a hundred times lower than that reported for HPLC. As for the previous example, this paper shows promising results with simple model solutions, far simpler than real biofluid samples, in which albumin and other proteins as well are already present.

Yuen et al. [72] reported the quantification of paclitaxel in blood plasma with the use of gold-polystyrene beads as SERS substrates and a multivariate calibration based on PLS. Polystyrene beads were deposited on a microscope glass slide by using a sedimentation technique, covered with a 15 nm-thick gold film and heated with microwaves. SERS spectra were measured at the concentration range from 1.0 × 10^−8^ M to 1.0 × 10^−7^ M, which according to the authors is comparable to the concentration detected by more time-consuming reference methods such as HPLC.

**Figure 2:**
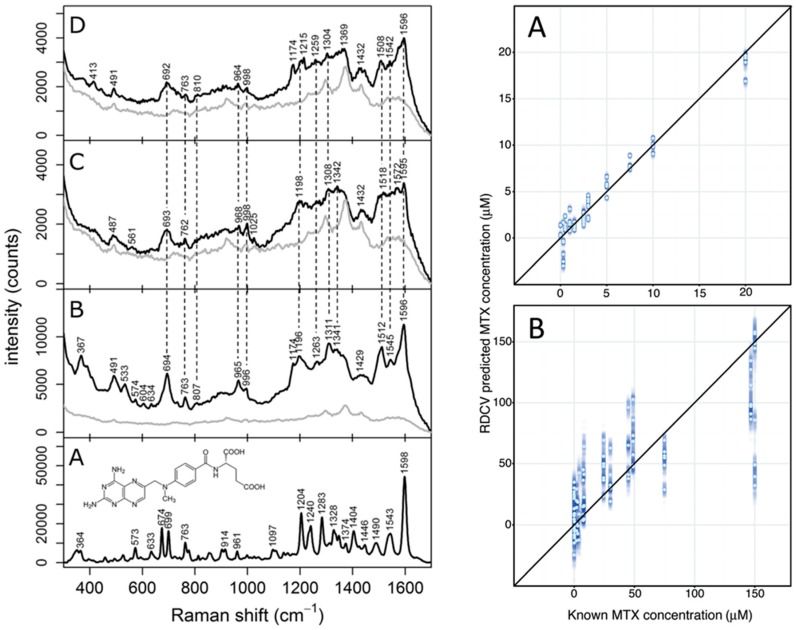
(**Left**) Raman of solid MTX (**A**) and SERS (**B**–**D**) spectra of MTX (10 µM) in PBS solution (**B**), PBS-HSA solution (**C**), and diluted human serum (**D**). Light gray lines represent the background SERS signal due to the substrate itself; (**Right**) diagnostic plots from repeated double cross-validation (RDCV) of SERS data collected from PBS-HSA solutions (**A**) and diluted human serum (**B**). Reprinted with permission from [54].

Nanoparticles deposited on filter paper are yet another example of a non-colloidal SERS substrate obtained with a bottom-up approach [54], which is possibly one of the less expensive substrates among the non-colloidal ones. MTX could be quantified in model solutions with HSA as well as spiked diluted human serum, resulting in a good linear SERS response in the concentration range of 1.0 × 10^−7^ M to 3.0 × 10^−4^ M, thus covering the therapeutic range of the drug (Figure 2).

Imatinib, another anti-cancer drug for which TDM is deemed useful, was successfully determined at concentrations of 0.1, 0.005, and 0.001 mg/mL (2 × 10^−6^ M–2 × 10^−4^ M) in plasma [71], using gold nanoparticles deposited on aluminum-covered glass slides as SERS substrates. This study was considered by the authors themselves as a “proof of concept”, and did not provide any calibration or other figures of merit (e.g., LOD, LOQ, etc.), but demonstrated that this drug could be observed with SERS at clinically relevant concentrations.

Graphene-modified metal nanostructures, already reported as colloidal substrates, were also used as non-colloidal substrates by Li et al. [76], who quantitatively determined MTX and some polar antibiotics in water in the range of 10^−9^ M–10^−3^ M using AgNPs—graphene nanocomposites deposited on disposable screen-printed electrodes. This paper exploited the polarity of these drugs to increase their concentration at the SERS substrates by varying the surface potential. The potential that was applied had a marked effect on both spectral shape and intensity, and with adequate potentials, MTX could be quantified with a LOD of 0.6 nM.

Flow systems, which were successfully used to control aggregation kinetics, thus improving repeatability, with colloidal substrates [45,80] can also be used with non-colloidal SERS substrates, provided that the methods allow for a substrate regeneration for multiple uses. In a recent work by Wu and Cunningham [77], 10 pharmaceutical compounds (including mitoxantrone) were measured in aqueous solutions using a flow-cell system. Plasmonic nanodome array structures, fabricated using a “nanoreplica molding process” on flexible plastic sheets, were used as SERS substrates. Upon flowing different solutions onto this substrate, several drugs could be detected at clinically relevant concentrations. SERS detection was shown to be almost reversible so that flushing the substrate with DI water caused the SERS signal to disappear almost entirely. This approach was demonstrated to be repeatable, and quantification was shown to be possible with a univariate approach, yielding a LOD of 18.6 ng/mL (~4.18 × 10^−8^ M) for mitoxantrone.

**Figure 3:**
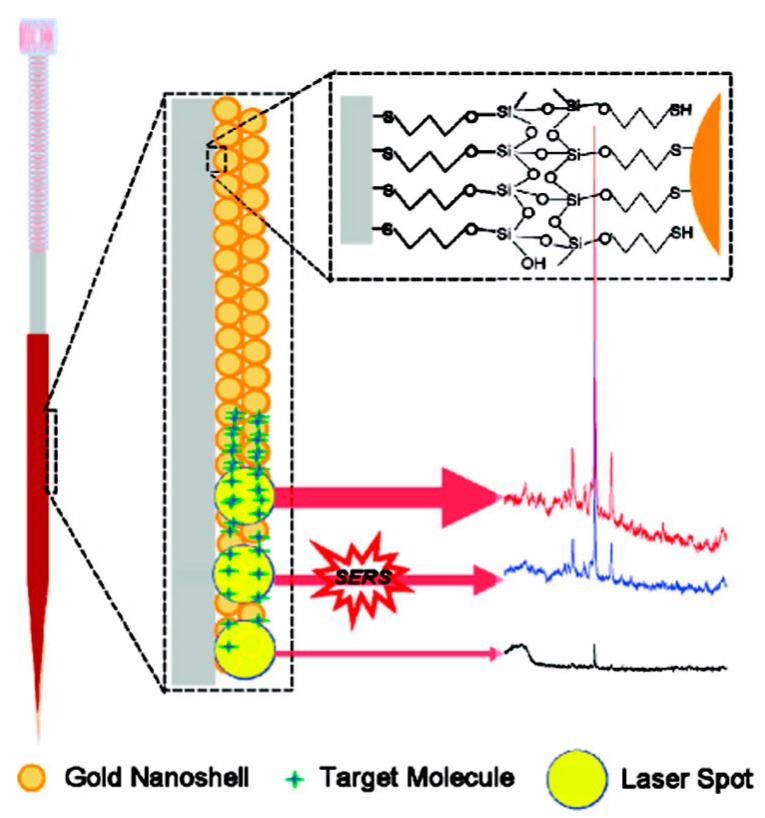
Illustration of the structure of a SERS-active needle and the SERS detection of a depth profile based on the SERS-active needle. Reprinted with permission from [69].

SERS detection and quantification of drugs were also achieved using innovative non-colloidal substrates, designed to be directly used as sample collection tools, with the aim of a faster and easier analysis. Sample collection or handling tools such as needles [69] or capillaries [67] were modified with metal nanoparticles to work as SERS substrates. The idea is very interesting since it could enable sample collection directly from the patient, allowing a direct SERS measurement. Up to now, the results reported in the literature are still preliminary, although very promising and worth developing. Dong et al. [69] reported the in vivo detection of 6-MP: SERS-active needles were inserted into a rabbit ear vein and *vastus lateralis* tendon for 10 min to detect the drug concentration in blood and muscles, respectively. The structure of the needle is shown in Figure 3. The main problem with this approach, so far, is that the quantitative analysis was problematic because of difficulties in controlling the volume of the blood in the needle. Despite the fact that no calibration curve or other FOMs were reported, this paper shows that a fast, real-time in vivo quantitative determination with SERS is feasible and that further investigation in this direction is certainly worth pursuing.

A similar approach with nanoparticle-modified capillaries was also reported with biofluids other than blood. Farquharson et al. [67] performed SERS measurements of 5-fluoro uracil (5-FU) in saliva with the use of SERS-active capillaries, internally coated with a layer of silver-doped sol–gel. The aim of the experiment was to reduce the time of the analysis (to 5 min) and the amount of saliva (<1 mL), with respect to other methods. With these SERS-active capillaries, it was possible to measure 5-FU at the concentration of 2 μg/mL in water and 500 μg/mL in saliva (1.54 × 10^−5^ M and 3.8 × 10^−3^ M, respectively). Again, the authors did not present any calibration plot, or any FOM (they estimated a “limit of detection”, but in the absence of a calibration). In spite of these methodological issues concerning quantification, since the toxicity of 5-FU was correlated with drug levels above 3 mg/L (2.3 × 10^−5^ M) [87], the methodology needs to be improved for a successful application in TDM.

### 2.3. Hybrid Substrates

In some cases, SERS detection was achieved using a so-called “sandwich” strategy, making use of two different substrates in a hybrid method, with the aim of trapping the analyte in between the different metal nanostructures [88]. In a recent work by Yang et al. [73], folic acid (FA) and MTX were trapped between a two layers of silver nanoparticles. To show the advantage of this sandwich strategy over common substrates, reference measurements were performed using silver nanoparticles only. With this approach it was possible to detect both FA and MTX in the presence of bovine serum albumin at a concentration of 10^−9^ M. Despite the fact that it was only demonstrated for simple model solutions, the detection of such a low concentration of a drug points to the potential of this approach in terms of sensitivity. On the other hand, such a method, involving the application of two different substrates at separate steps, appears to be more complicated to apply, making it less suitable for point-of-care tests.

## 3. Conclusions and Perspectives

Although in recent years an increase in the number of papers in this field has been observed, publications about SERS applied to TDM are still relatively few, and no definite methodological trend is present. In our opinion, this is a direct consequence of the many difficulties and challenges encountered when the quantification of drugs is pursued with SERS in complex samples such as biofluids.

The studies published so far clearly demonstrate the potential of SERS for TDM, as many of them reported the feasibility of quantification at clinically-relevant concentrations, but most of these studies were carried out in model solutions, far simpler than real biofluids such as plasma or serum. In fact, only one example of the application to real clinical samples (i.e., serum of patients treated with mitoxantrone) has been published, and SERS showed an analytical performance comparable to HPLC [45]. Other drugs for which TDM is deemed relevant have been successfully quantified only in spiked biofluids (6-mercaptopurine [69], doxorubicin [70], imatinib [71], methotrexate [54], mitoxantrone [45], and paclitaxel [72]). All of the other studies considered buffered solutions of drugs, sometimes in the presence of serum proteins such as HSA. The role and impact of the chemical complexity of biofluids on SERS have been already addressed in Section 1.3, and, likely, more fundamental and methodological studies on biofluid-metal interactions are needed to better tackle this issue.

Thus, in spite of its potential to advance individualized care and reduce the associated healthcare costs, SERS technology has not yet reached the maturity for routine analysis, and several key challenges need to be addressed before it can be adopted in clinical practice.

**Figure 4:**
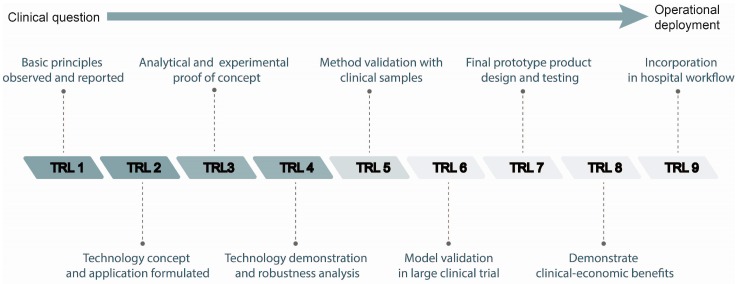
Roadmap for the clinical translation of SERS for TDM. The well-known technology readiness levels (TRL) reached by the SERS works reported in this review are indicated with a color intensity scale: only the first 5 levels are covered. The majority of studies are at the “proof of principle” level or the “validation of single functionality” level (TRL 3–TRL4).

Figure 4 schematically depicts the roadmap for a clinical translation process, including the Technology Readiness Levels (TRL) that are typically used to assess the maturity of novel technologies [89]. Current research for the implementation of SERS as a tool for TDM is at an early stage, as most of the studies have been focused on substrate development and evaluation in simple solvents/surrogate matrices (TRL 2–3 and 4), with only one study mentioned above reporting the analysis of real samples (TRL 5). Although more studies on real samples are needed for the use in clinical practice (TRL 9), progress has been substantial.

The foremost challenge, which is a general problem in SERS going beyond TDM applications, is the development of “standardized” substrates (and thus of standard definitions, guidelines, and protocols) that, with adequate adaptations, can be generally used for the detection of different molecules/groups of molecules. For TDM, which involves quantitative analysis, this aspect is even more crucial than for other applications. So far, each research group has used its own SERS substrates, prepared according to different protocols: only in a few publications was the same substrate applied to more than one drug. The availability of commercial, repeatable, and standardized SERS substrates at reasonable prices is thus a pre-requisite for the application of SERS outside academic and institutional research centers. We believe this is particularly true for clinical applications, which are strictly regulated by national laws.

A second challenge is, as already mentioned, the intrinsic chemical complexity of biofluids, whose components often heavily interfere with the SERS analysis. The sequestration into bulk solution of a relevant fraction of the drug and the competition with biofluid constituents for the metal surface are two of the most critical problems to be solved, either by efficient and fast de-proteinization and separation steps or by surface functionalization with selective recognition elements. Both strategies should aim for the simplification of the biofluid complexity at the surface of the SERS substrate.

A third challenge involves the correct use of data analysis and calibration methods, as well as a proper estimation of important FOMs, such as LODs and LOQs. In fact, for the majority of the literature considered in this review, FOMs are often reported only partially and/or are not properly defined. As an example of this, only one of the considered studies used the official IUPAC definition for the LOD calculation, while nine out of fifteen studies did not even report the LOD for the method (Table 2). This is especially important in view of clinical applications, where analytical methods should comply with formal guidelines. Moreover, the potential of multivariate calibration methods, which are especially useful in recognizing the SERS signal of the analyte from the fluctuations due to the interindividual variability intrinsic to biofluids, has not yet been fully exploited.

Last but not least, a SERS-based method for TDM should be developed that considers several characteristics required for a routine use in a clinical setting, such as robustness (i.e., it should not involve too delicate or complex instrumentation), cost (it should be competitive with respect to the available methods), ease of use (it should be easy to operate by non-specialized users) and ease of the interpretation of results (it should give a response in terms of concentration and error). Many papers, even those that presented very promising results, sometimes did not consider or discuss these issues.

## Figures and Tables

**Table 1:** Pros and cons of analytical techniques currently used in TDM, compared with SERS.

Technique	Description	Pros	Cons
*Chromatography-based methods*	Chromatography separates individual compounds by their physical or chemical interaction with an immobile material. Once separated, combined selective MS techniques provide mass-based identification of the compounds	Gold standard; Robust methods with superior sensitivity and sensibility; Relatively free from interferences; Multiplexing capabilities; Reduced drug class/metabolites cross reactivity	Time consuming; Laboratory-developed tests; Interlaboratory variability; Matrix effects; High technical expertise required; High costs for installation, personnel training, and method validation; Need for sample clean-up
GC-MS/MS LC-MS/MS			
*Immunoassay platforms*	Analyte is detected by its binding with a specific binding molecule, which in most cases is an analyte-specific antibody	Small amount of sample (<100 μL); Run on automated, continuous, random access systems; No need for sample clean-up; Multiplexing capabilities	Several steps to achieve quantification of the analyte; Reduced specificity and sensitivity; Often show significant bias; Antibody cross-reactivity; Interferences from bilirubin, hemoglobin, high lipid content, very high or very low protein content, endogenous antibodies, various drugs and metabolites
ACMIA, CEDIA, CMIA, ELISA, EMIT, FPIA, MEIA, PETNIA			
*SERS-based methods*	Inelastic light scattering on molecule adsorbed on the roughened metal surface is measured	No need for sample preparation; Fast measurement; Multiplexing capabilities; Availability of portable Raman spectrometer	Very often high RSD of the SERS substrates; method optimization needed for each drug;

GC-MS/MS, Gas Chromatography-tandem Mass Spectrometry; LC-MS/MS, Liquid Chromatography-tandem Mass Spectrometry; ACMIA, Antibody Conjugated Magnetic Immunoassay; CEDIA, Cloned Enzyme Donor Immunoassay; CMIA, Chemiluminescent Microparticle Immunoassay; ELISA, Enzyme-linked Immunosorbent Assay; EMIT, Enzyme Multiplied Immunoassay Technique; FPIA, Fluorescence Polarization Immunoassay; MEIA, Microparticle Enzyme Immunoassay; PETNIA, Particle-Enhanced Turbidimetric Inhibition Immunoassay; RSD, Relative Standard Deviation.

**Table 2:** Overview of research involving SERS for TDM of various type of chemotherapeutic drugs.

Sample	Drug	SERS Substrate	Laser Line (nm)	LOD (M)	Calibration	Refs.
*Clinical sample*						
serum	Mitoxantrone	Ag colloid FLOW	514 633	4 × 10^−11 b^	U	[45]
*Spiked body fluid*						
saliva	5-FU	SERS-active capillaries	785	1.15 × 10^−06 a^	U	[67,68]
blood	6-MP	Si-AuNPs needles	785	n.r.	n.r.	[69]
bovine plasma	Doxorubicin	Ag colloid	488	n.r.	M	[70]
plasma	Imatinib	Au on glass with Al	785	n.r.	n.r	[71]
human serum	MTX	Au colloid on paper	785	n.r.	M	[54]
blood plasma	Paclitaxel	Au-polystyrene beads	785	n.r.	M	[72]
*Surrogate matrix*						
1.5% HSA-PBS	Irinotecan	Ag and Au colloid on TLC plate	514 785	n.r.	n.r.	[40]
1% BSA-PBS	MTX	Sandwich substrate	532	10^−09 b^	n.r.	[73]
0.6% HSA	Paclitaxel	Ag colloid	532	n.r.	n.r.	[74]
*Other solutions*						
water	6-MP	β-CD AgNPs Ag colloid	785	2.4 × 10^−09 a^ 1.4 × 10^−08 a^	U	[75]
water	MTX	Ag-graphene	785	6 × 10^−10 b^	U	[76]
water	Imatinib	Au on glass with Al	785	n.r.	n.r.	[71]
water	Mitoxantrone	PNA	785	4.18 × 10^−08 c^	U	[77]
BRB (pH 2.0)	6-MP	GO/AgNP hybrids	532	1.05 × 10^−07 b^	U	[78]
MeOH	Irinotecan SN-38 Sunitinib	Klarite™	633	34–40 *^,e^ 11–28 *^,e^ 11–15 *^,e^	U	[79]
150 nM KOH	MTX	Ag colloid FLOW CELL	514 785	1.70 × 10^−07 b^	U	[80]
DMSO	Paclitaxel	GNC UV-NIL	633	10^−09 f^	U	[81]

β-CD—β-Cyclodextrin; 5-FU—5-fluorouracile; 6-MP—6-mercaptopurin; BRB—Britton-Robinson Buffer; BSA—bovine serum albumin; GNC—gold nanocylinder; GO/MNP—graphene oxide/silver nanoparticles; HAS—human serum albumin; KOH—potassium hydroxide; M—multivariate; MTX—Methotrexate; n.r.—not reported; PNA—plasmonic nanodome array; U—univariate. * ng/mm^2^; ^a^ LOD is three times the signal-to-noise ratio; ^b^ LOD calculation not defined; ^c^ LOD is three times the standard deviation of five blank intensities; ^d^ LOD is three times the background noise; ^e^ LOD is the lower amount of analyte clearly identified above the noise; ^f^ LOD is 3σ/k with σ as the standard deviation of blank measures and k as the slope of the calibration plot (IUPAC).

**Table 3:** Summary of HPLC and SERS* Analysis Results for Patient Samples, reprinted with permission from ref. [45].

	concentration of mitoxantrone (ng/mL)	
Time	HPLC	SERS *
0	0	nd
5	3.1	2.9
10	1.9	1.0
15	252.3	247.3
20	186.9	183.2
30	53.7	54.1
45	23.7	20.3
60	11.6	9.2
90	9.4	8.9
120	6.2	6.0
180	5.7	4.8
240	1.8	1.0
360	3.2	3.0
720	1.8	1.3

* In this study, authors chose a laser frequency which is in resonance with the analyte, so they correctly defined it as surface enhanced resonance Raman spectroscopy (SERRS) instead of SERS.
